# Coastal methane emissions driven by aerotolerant methanogens using seaweed and seagrass metabolites

**DOI:** 10.1038/s41561-025-01768-3

**Published:** 2025-08-07

**Authors:** N. Hall, W. W. Wong, R. Lappan, F. Ricci, K. J. Jeppe, R. N. Glud, S. Kawaichi, A-E. Rotaru, C. Greening, P. L. M. Cook

**Affiliations:** 1 Water Studies, School of Chemistry, Monash University, Melbourne, Victoria Australia; 2 Department of Microbiology, Biomedicine Discovery Institute, Monash University, Melbourne, Victoria Australia; 3 Securing Antarctica’s Environmental Future, Monash University, Melbourne, Victoria Australia; 4 Monash Proteomics and Metabolomics Platform, Department of Biochemistry and Molecular Biology, Biomedicine Discovery Institute, Monash University, Melbourne, Victoria Australia; 5 Nordcee, Department of Biology, University of Southern Denmark, Odense, Denmark; 6 HADAL, DIAS, University of Southern Denmark, Odense, Denmark; 7 Department of Ocean and Environmental Sciences, Tokyo University of Marine Science and Technology, Tokyo, Japan

**Keywords:** Carbon cycle, Marine biology

## Abstract

Methanogenesis is thought to be limited to strictly anoxic environments. While oxygenated oceans are a known methane source, it is argued that methane production is driven by methylphosphonate-degrading bacteria and potentially other sources rather than by methanogenic archaea. Here we develop in situ monitoring and ex situ manipulation experiments, combined with biogeochemical, metagenomic and culture-based experiments, to show that methane is rapidly produced by archaea in frequently oxygenated sandy sediments. We show that methane emissions from sandy sediments are not inhibited by repeated oxygen exposure and suggest the activity is driven by aerotolerant methylotrophic methanogens (primarily Methanosarcinaceae) broadly distributed in the surface layers of sandy sediments. Moreover, we show that methane emissions are driven by methylated seaweed and seagrass metabolites, revealing a feedback loop between primary production and greenhouse gas emissions.

## Abstract (web summary)

Experiments suggest aerotolerant archaea produce methane in the surface layers of coastal sandy sediments and that this activity is driven by seaweed and seagrass metabolites.

## Main

Methanogens are typically considered to be strict anaerobes, highly sensitive to oxygen and therefore restricted to stable anoxic environments^1,2^. While some methanogens have been shown to survive periods of oxygen exposure^3^, active methanogenesis in the environment recovers over timescales of weeks to months^4–6^.

Of total marine emissions, the contribution of near-shore shallow coastal areas is both the largest and the most uncertain, estimated to constitute approximately 75% of marine methane emissions globally, offsetting much of the CO_2_ drawdown of these highly productive ecosystems^7^. While the methane emissions of mangroves, salt marshes and other vegetated coastal environments have been extensively studied^8–12^, permeable (sandy) coasts have been largely overlooked, despite covering 50% of the world’s continental margins^13^.

Methane supersaturation is frequently observed in near-shore waters overlying permeable sediments and has generally been explained by the input of methane-rich groundwater or riverine water, or the seepage of methane from below the sulfate–methane transition zone^14–16^. It has also been proposed that excess methane in these zones could be produced by aerobic bacteria during methylphosphonate degradation^17,18^ or through unresolved processes in phytoplankton^19–23^. Archaeal methanogenesis has been disregarded as a substantial contributor given that coastal permeable sediments are characterized by sudden and unpredictable changes in redox conditions and high sulfate concentrations^24,25^. These characteristics promote the dominance of metabolically flexible facultative anaerobic bacteria^26^ and are thought to exclude methanogenic archaea.

Here, we demonstrate that methylotrophic methanogenesis is active in frequently oxygenated surface sediments and is stimulated by seaweed and seagrass (collectively macrophyte) metabolites. By pairing the isolation of two strains of *Methanococcoides* sp. with metagenomic profiling, we show that archaeal methylotrophic methanogens are widespread in sandy sediments at sites in both Australia and Europe.

## Methane produced in shallow oxygenated sediments

**Fig. 1:**
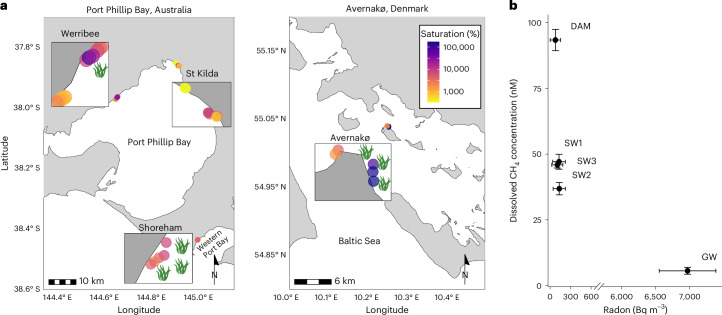
High surface water methane saturation and low radon over sandy beaches. **a**, A map of the measured in situ surface water methane supersaturation at Australian and Danish sites. The green seaweed symbols indicate sites with high macrophyte accumulation. For detailed site information see Supplementary Table 1. Australian and Danish coastline shapefiles were downloaded from the Science Education Resource Center^74^ and the European Environment Agency^75^, respectively. **b**, Methane (nM) versus radon (Bq m^−3^) concentrations in groundwater (GW), surface water clear of drift algae (SW) and surface water from within a drift algal mat (DAM) at Werribee. The error bars indicate 1 s.d. from the mean, *n* = 3 (methane) or value and error given by a ^222^Radon analyser.

Methane concentrations were measured in near-shore surface waters (water column depth 20–70 cm) over beaches around Port Phillip Bay and Westernport Bay (Australia) and Avernakø (Denmark). Methane was consistently oversaturated with respect to the atmosphere, though the extent of saturation varied over four orders of magnitude, with values ranging from 380 ± 30% to 189,000 ± 7,000% saturated with respect to the atmosphere (Fig. 1a and Supplementary Table 2). No relationship was found between methane and radon concentrations (Fig. 1b), indicating methane was not sourced from groundwater, contrasting with similar previously described cases^27,28^. Higher methane concentrations were observed over local (metres to tens of metres) scales adjacent to, or within, seaweed and seagrass mats at both Werribee (Fig. 1b) and Avernakø (Fig. 1a), indicating that this biomass enhances methane production in the environment. Macrophyte accumulation is often associated with lower energy sites, which enhance microbial activity and lower oxygen penetration in the sediment. However, hydrodynamic exposure alone does not explain patterns of methane concentrations observed in situ; for example, Shoreham is much more exposed than the St Kilda site, which is protected by a pier and boat harbour. In addition, other sites with very similar levels of exposure but different local macrophyte accumulation, such as Avernakø East and West and Werribee beaches, showed orders of magnitude different surface water methane concentrations.

**Fig. 2:**
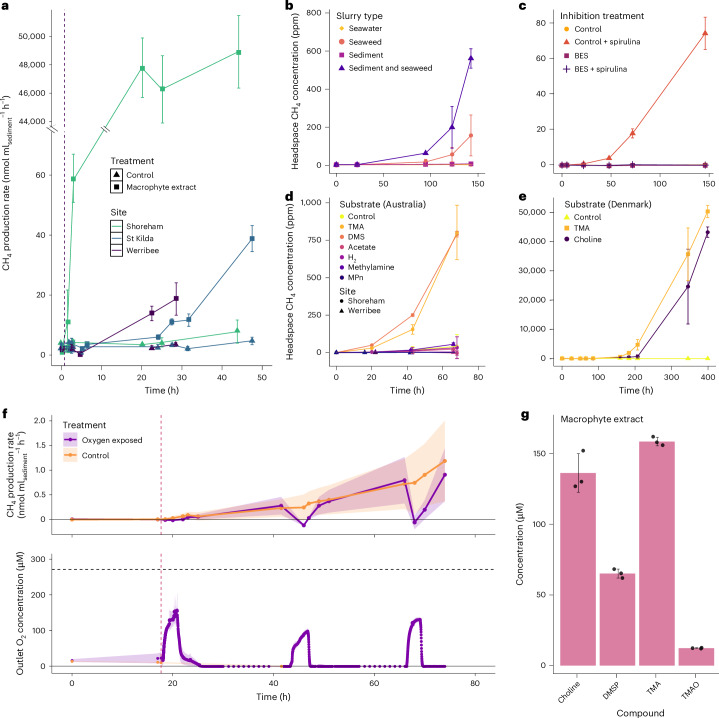
Laboratory incubations show source of methane from sediments to be oxygen-tolerant methylotrophic methanogens. **a**, FTR experiments show methane production after the onset of anoxia and macrophyte extract addition (dashed line) in surface sediments (0–5 cm) from a site with macrophyte accumulation (Shoreham) and sites without accumulation (Werribee and St Kilda). The error bars represent the s.d. from the mean, *n* = 4. Note the broken *y* axis. **b**, Methane production in slurry incubations of seawater, sediment and seaweed (brown drift algae) from Werribee. **c**, Methane production in Werribee sediment slurry incubations with spirulina additions with and without the specific archaeal methanogenesis inhibitor BES (20 mM). **d**, Methane production in sediment slurry incubations with the addition of 100 µM concentrations of TMA, DMS, acetate, hydrogen (340 ± 20 ppm), methylamine and MPn in sediments from Shoreham and Werribee. **e**, Methane production in sediment slurry incubations with addition of 100 µM concentrations of TMA and choline in sediments from Avernakø, Denmark. All slurries were prepared with surface sediment (0–5 cm) and argon purged at the start (time, 0 h). The error bars of all slurry experiments represent the s.d. from the mean of three independent slurries. **f**, Methane production after multiple oxygenation events in an FTR (Shoreham sediment, 0–5 cm). The vertical dashed line indicates macrophyte extract addition and the horizontal black line indicates 100% air saturation. The error bars represent the s.d. from the mean, *n* = 4. **g**, Methylated metabolite concentration in macrophyte extract samples. The error bars represent 1 s.d. from the mean, *n* = 3.

To determine the local site of methane production, rates were compared between slurry incubations with combinations of surface sediment (0–5 cm), seawater and seaweed. The combination of sediment and seaweed stimulated the highest rates of methane production, followed by seaweed only (Fig. 2b), whereas no methane production was detectable in seawater-only controls or sediment only. This indicates that microbes responsible for methane production are primarily located in sediment but benefit from substrates derived from seaweed or seagrass.

Surface sediments were studied given that they are exposed to the highest levels of dissolved metabolites from macrophyte biomass and we hypothesized that they may host aerotolerant methane producers. The sands were sampled from the shallow (<1 m water depth) intertidal zone, where bottom-water dissolved oxygen was consistently ~100% air saturation and waves generate the dominant boundary-layer flows^29,30^. The depth cut off was 5 cm as this was above the visibly reduced iron sulfide layer in all sampled sediments, indicating relatively frequent oxygen exposure^31^. Oxygen profiles in the sediment were measured at St Kilda and Werribee, confirming that oxygen penetrates frequently to at least 5 cm even in the most protected Australian sites (Supplementary Fig. 1). These findings showed, surprisingly, that frequently oxygenated surface sediments had strong methanogenic potential.

## Methylotrophic archaea dominate emissions

We initially hypothesized that bacteria or microalgae, rather than methanogenic archaea, may be responsible for methane production in permeable sediments because frequent oxygenation of the surface sand would inhibit archaeal methanogenesis^32^. 2-bromoethane sulfonate (BES) was used as a targeted inhibitor of archaeal methanogenesis through its inhibition of methyl-CoM reductase, the terminal enzyme in all known pathways of archaeal methanogenesis^33,34^, the addition of which completely inhibited methane production in our slurries (Fig. 2b). Methylphosphonates did not stimulate methane production in either slurries (Fig. 2e) or oxic or anoxic flow-through reactors (FTRs) (Supplementary Fig. 2), ruling out methylphosphonate degradation as a major source of methane in these environments, which is unsurprising given these waters are unlikely to be phosphate limited. Another possible pathway of bacterial methane production is via certain aspartate aminotransferases^19^, yet the lack of any measurable methane production in slurries amended with BES indicates that this pathway also does not constitute a major source in shallow sands. By experimentally ruling out other known pathways of methane production in these sands and showing clear production only in the absence of BES, we conclude that the primary source of methane in the targeted intertidal permeable sediments is archaeal methanogenesis, distinct from previous explanations of the oceanic methane paradox in marine surface waters^35–37^.

We investigated acetoclastic, hydrogenotrophic and methylotrophic methanogenesis pathways using targeted substrate addition (Fig. 2d) and found that methylotrophic methanogenesis predominated, as indicated by the methane production above the control only with addition of methylated substrates trimethylamine (TMA), methylamine and dimethyl sulfide (DMS), but not acetate, hydrogen, formate or methylphosphonate. As many organisms, such as sulfate and nitrate reducers, outcompete methanogens for acetate and hydrogen but not for methylated substrates, methylotrophic methanogenesis can occur in unreduced environments with abundant alternative electron acceptors^38,39^. In addition, surface sediments are often exposed to high fluxes of labile methylated compounds and may therefore be adapted to quickly utilize these substrates, as described in seagrass meadows^40^. Methylated compounds are abundant in coastal regions and are formed through the breakdown of widely occurring macrophyte osmolytes such as glycine betaine, choline and dimethyl sulfoniopropionate (DMSP)^41,42^. These dissolved metabolites are quickly metabolized in sediment, therefore no methane production was observed in sand-only experiments.

It is important to note that while slurry experiments are useful to test the ability of the microbial community to use specific substrates, as they are a closed and highly manipulated environment, they do not closely resemble in situ conditions. This is especially the case in permeable sediments where advective flow is an important factor for interstitial solute transport and rate measurements.

To address this limitation, we compared community composition and priming effects in sediments from different sites with different levels of macrophyte accumulation using FTR experiments. FTRs more realistically emulate the advective flow, which is caused by wave and tidal pumping in surface permeable sediments^43,44^ and allow continuous renewal of substrates and prevent build up of reaction products. Remarkably, methanogenesis started within ~20 h of the transition to anoxia and addition of macrophyte extract in FTRs from all Australian sites (Fig. 2a)^4–6^, regardless of site. However, the rate of methane production at the site with the most macrophyte accumulation (Shoreham) was both faster (within 1.5 h) and four orders of magnitude higher after 20 h than the two sites with less accumulation. This indicates that permeable sediments may generally harbour the latent potential for methanogenesis, but indicates that macrophyte accumulation increases abundance and/or activity of methanogens. The macrophyte extract used for FTR experiments was analysed for methylated metabolites TMA, choline, trimethylamine *N*-oxide (TMAO), DMS and DMSP. All were detected in the macrophyte extract, except DMS (Fig. 2g), and the sum of these metabolites (correcting for the stoichiometric number of methyl groups per molecule) accounted for 86% of maximum methane production in the Shoreham FTRs. The remaining 14% of the methane production may be accounted for by other methylated compounds or the breakdown of larger molecules.

While methane production rates are difficult to compare between different sediment types owing to the differences in advective and diffusive flux, some comparisons can be made between our experimental rates to place them in the context of more well-studied environmental fluxes. If integrated conservatively over a 0.5 cm permeable sediment depth as the depth of advective penetration of high-substrate surface water in the intertidal zone^45^, the maximum methane production rate at Shoreham is approximately 3.8 g m^−2^ h^−1^, nearly three orders of magnitude higher than flux rates recently reported for tropical wetlands^46,47^. The CH_4_:CO_2_ carbon remineralization ratio reached 1:9 after 44 h (Supplementary Fig. 3), exceeding the typical ratio for most types of wetland sediment and indicating that methanogenesis may be a quantitatively important carbon remineralization path in permeable sediments^48^. These findings highlight not only the ability of methanogenic archaea to adapt to highly dynamic environments and recover extremely quickly after oxygen exposure, but also that their methane production rates can be comparable to, or even exceed, those of the most active methanogenic environments studied^48–50^.

To investigate the effect of repeated oxygen exposure as is believed to occur in situ, another FTR experiment was conducted with a 3 h period of oxygen exposure (approximately 40–60% air saturation at the outlet and 100% at the inlet) every 24 h for three consecutive days (Fig. 2f). Remarkably, methanogenesis resumed within 1–2 h of the return to anoxia after every oxygen exposure, showing that recovery is not affected by repeated exposure. Furthermore, the growth of methanogens stimulated by the addition of macrophyte extract, was not inhibited in the oxygen-exposed FTRs, compared with the constantly anoxic control, as inferred from increasing methane production rates. This contrasts strongly with previous studies that showed methane emissions from sediments undergoing an oxic–anoxic transition (for example, rice paddies or flooded soils) to begin only on the order of weeks to months^4–6^. In addition, while studies have shown that methanogenesis can occur in oxygenated soils^51,52^, these studies proposed that this phenomenon is explained by anoxic microniches rather than the oxygen tolerance of methanogens. We therefore determined to isolate methanogens from permeable sediments to study their abilities to recover from oxygen exposure in culture, independent of their environment.

## Metagenomes and novel isolates confirm aerotolerant methanogens

**Fig. 3:**
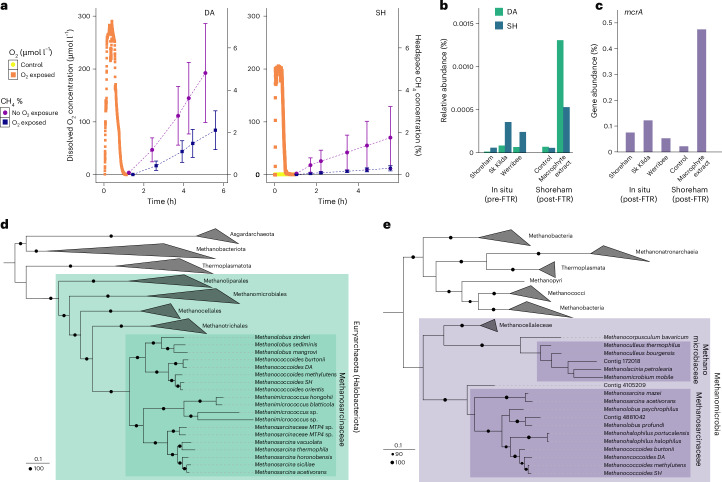
Cultured isolate experiments and genomes as well as sediment metagenome data confirm activity and distribution of methanogens. **a**, Methane production after oxygen exposure in sand isolates *Methanococcoides* sp. DA and SH. The error bars represent the s.d. from the mean, SH *n* = 2 and DA *n* = 3. **b**, Read mapping of metagenomic reads to isolate sequences from in situ and post-experiment sediment samples (the methane production results from the same sediment samples are shown in Fig. 2a). **c**, *mcrA* gene abundance in all metagenome samples. **d**, A maximum-likelihood phylogenetic genome tree showing both isolates (bold) and reference genomes. Amino acid sequences from the isolated genomes were aligned with reference genomes. The coloured boxes indicate family (dark shade) and phylum (light shade). The legends for tree scale and bootstrap of 100 are reported in the bottom-left corner. **e**, A maximum-likelihood phylogenetic gene tree of *mcrA* sequences, displaying isolates (bold), contigs from metagenomic analyses of in situ and post-experiment sediment samples (bold) and reference sequences. Amino acid sequences of isolates and contigs were aligned with reference sequences. The coloured boxes indicate family (dark shade) and class (light shade). The legends for tree scale and bootstrap of 90 and 100 are reported in the bottom-left corner. Sequence alignments used to generate these phylogenetic trees are provided and source trees are available in tree file format.

Methanogens were isolated from both Australian and Danish sites to investigate the aerotolerance (the ability of obligate anaerobes to survive and recover metabolic functions after exposure to oxygen) of methanogens from permeable sediments, independent of potential anaerobic sand grain microniches caused by bacterial oxygen consumption. To select for aerotolerant methylotrophic methanogens, surface sands (0–5 cm) were collected and incubated with TMA to increase the abundance of methylotrophic methanogens before isolation. Two methanogens were isolated, from Avernakø, Denmark (strain DA) and Shoreham, Australia (strain SH), both from the genus *Methanococcoides* (family Methanosarcinaceae). When compared with all representative genomes of *Methanococcoides*, DA was most closely related to *Methanococcoides burtonii* (average nucleotide identity (ANI) value of 89.3) and SH to *Methanococcoides orientis* (ANI value of 90.0). Between the two isolates, the ANI value was 80.3. In support of the biogeochemical observations, both methanogens rapidly produced methane in the presence of TMA. Moreover, activity of these methanogens resumed immediately following transient (approximately 30 min) exposure to 200–300 µmol l^−1^ oxygen (Fig. 3a). Interestingly, these methanogens sometimes formed clumps or biofilms after exposure to oxygen, though this was not uniformly the case (Supplementary information).

Analyses of the genome sequences of both isolates revealed remarkable similarities in their methanogenesis pathways and antioxidant systems, despite being isolated from geographically and climatically distinct locations, suggesting that these traits are important for adaptation in sandy sediments. Both isolates encoded a complete methylotrophic methanogenesis pathway with methyltransferases for tri-, di- and monomethylamines and methanol (*mttABC*, *mtbABC*, *mtmABC* and *mtaABC*, respectively). No methylthiol methyltransferases (*mts*) were found, despite DMS stimulating methane production similarly to TMA in slurries^53^. It has been proposed that some methanogens may use *mtt* and *mta* methyltransferases to metabolize DMS^53^. Both genomes encode genes involved in oxidative stress protection, including the F_420_H_2_-dependent oxidase that detoxifies O_2_ to water and is unique to methanogens^54^, as well as those typically associated with aerobic organisms such as superoxide reductase and catalase-peroxidase^55^. In addition, both genomes encoded a variety of other proteins with potential antioxidant roles such as rubredoxins^56^, thioredoxins^57^ and peroxiredoxins^3,58,59^. While these antioxidant genes have been previously identified in many methanogens^57^, this study gives robust experimental evidence to previous genomic predictions, showing the fastest recovery times after oxygen exposure of any methanogen studied so far.

Shotgun metagenomics was used to determine whether methanogens and their functional genes were present in the permeable sediments. Samples for analysis were collected at the beginning of the FTR experiments for all sites, as well as at the end of the experiment for the Shoreham samples. While no methanogen metagenome assembled genomes were recovered (probably due to their low initial biomass), read mapping of metagenomic reads to the isolate genomes revealed that both strains were present in the in situ samples from all sites and their abundance increased 9–10-fold with the macrophyte extract treatment in FTRs (Fig. 3b). The marker gene for archaeal methanogenesis, *mcrA* (encoding methyl-CoM reductase), was also found in samples from all sites (Fig. 3c). This gene was encoded by a small proportion of the community (0.023–0.476%) in line with previous observations for surface sands^26,60,61^; however, it is well established that methanogens are highly transcriptionally and biogeochemically active, even in niches where their abundance is low^11^. Furthermore, *mcrA* gene abundance increased sixfold in the Shoreham FTR experiment with macrophyte extract but not in the control, indicating the growth of methanogens under experimental conditions. Most sequences were affiliated with Methanosarcinaceae, especially *Methanolobus, Methanococcoides* and *Methanosarcina* species (Supplementary Table 5), all of which are known to utilize methylated substrates^39^, giving a genetic basis for the experimental finding that all sites harbour the latent potential for methylotrophic methanogenesis, with growth under high-metabolite conditions. Genes for bacterial methane production pathways were also found in low abundance (C-P lyase *phnJ*, aspartate aminotransferase *aat*; Supplementary Table 4), indicating other microbes and pathways may also produce methane in these sediments; however, the collective in situ observations, ex situ inhibition and stimulation studies and culture-based profiling all suggest methylotrophic methanogenesis is the dominant pathway and that the others are negligible.

## Climate consequences in a changing world

The evidence presented here shows the activity of the most oxygen-tolerant methanogens described so far, both in whole-community and isolate settings. Combined with high rate measurements and evidence of macrophyte biomass being the primary driving factor, this redefines the range of environments that can be described as highly methanogenic and suggests climate consequences to changes in coastal permeable environments, which have not been previously considered.

While this study does not attempt to systematically quantify the methane fluxes that could be attributed to this pathway, we calculated the average as well as the upper and lower bounds of flux rates at the Australian sites based on in situ supersaturation measurements to contextualize these results. Our calculated fluxes spanned four orders of magnitude, depending on the water oversaturation and wind speed, with a minimum of 0.2 mg m^−2^ day^−1^, an average flux estimate of 23 mg m^−2^ day^−1^ and a maximum 540 mg m^−2^ day^−1^. These values were calculated using Q1, Q2 and Q3 wind speeds and the lowest, average and highest in situ concentrations, respectively, and the results emphasize how locally variable and complex near-shore surface water fluxes are. The lower end of the range calculated here is comparable to the highest reported values in ref. ^7^. The average flux fell within the range of fluxes from mangroves and salt marshes, but higher than those reported for seagrass ecosystems reported in ref. ^62^ and higher than those reported in ref. ^63^. More extensive sampling across sites varying in macrophyte accumulation, across seasons and across conditions is needed to constrain the fluxes resulting from this pathway.

The shallow and turbulent nature of waters overlying coastal permeable sediments, combined with advective transport in the sediments, gives the study additional importance. In deeper waters and cohesive sediments, the balance of methanogenesis and methanotrophy is such that in the bulk of the ocean, the volume is undersaturated in methane with respect to the atmosphere^64^. However, in rippled permeable sediments, the redox seal is broken, with flow from reduced reaction zones exported directly through ripple peaks or lee sides depending on bedform and flow interactions^29,65^. This allows methane produced in shallow anoxic regions to reach the shallow overlying water where low residence times and high turbulence causes high rates of export to the atmosphere^14,66^. Therefore, the contribution of methane production in shallow permeable sediments to total marine methane emissions is probably disproportionately large.

The link between macrophyte biomass and methane emissions may cause seasonal patterns related to the different growth and decay phases of various seaweeds and seagrasses^67,68^. Therefore, local differences in species composition and growth drivers (for example, nutrient input, water temperature, impact of grazing or light availability as the primary driver) may cause large differences between sites, and seasonal drivers may be particularly distinct between tropical and temperate climates, which should be investigated in further detail.

Apart from seasonal changes, this study has particular consequence in the context of increasing eutrophication and rising sea temperatures, which are linked to increased algal growth and the frequency of large algal blooms in coastal zones^69–72^. Here, we have shown that deposition of this excess algal biomass on sandy coasts may result in increasingly large and frequent pulses of methane to the atmosphere and should be accounted for in future marine methane budgets and modelling. In particular, we note that many studies that quantify the net carbon sink/source dynamics of vegetated ecosystems focus on the sites where these macrophytes grow^73^, and we suggest that future work should focus on the mobility of degrading biomass and its potential greenhouse gas emissions when deposited in different ecosystems. As well as unintentional excess macrophyte growth caused by eutrophication, the results of this study further complicate CO_2_ removal by macrophytes, seagrasses or ‘blue carbon’ as a climate change mitigation strategy^7^, as enhanced methane emissions may offset much of the CO_2_ removal by these ecosystems.

## Methods

### Field measurements and sampling sites

In situ measurements and sediment sampling were conducted in Australia at Werribee, St Kilda and Shoreham. In Denmark, samples were collected from Avernakø. For the coordinates and dates of sampling, see Supplementary Table 1. These sites were selected for their different macrophyte accumulation characteristics. For details of each site, see Supplementary Fig. 5 (photos of each site) and Supplementary Tables 1 and 2 (descriptions, coordinates and measured methane concentrations).

Seawater samples were collected for in situ dissolved methane (CH_4_) analysis and were collected with minimal gas loss by over-filling 12 ml exetainers from the bottom up using syringes fit with 10 cm low gas-permeability tubing (MasterFlex 06401-16). Samples were preserved by adding 50 µl saturated HgCl_2_ and then stored at room temperature. For dissolved methane concentration, samples were analysed by gas chromatography interfaced with a pulsed-discharge helium ionization detector (GC–PDHID). For analysis details, see ‘Chemical analysis’ section below.

To determine grain size distribution, sand was collected using acrylic cores from the shallow subtidal zone and transported back to the laboratory. The top 0–5 cm were sectioned, pooled, dried and 500 g from each site used for analysis. Sand was progressively separated using ten sieves of different sizes (2, 1.204, 1, 0.85, 0.5, 0.15, 0.125, 0.08, 0.063 and 0.038 mm). Median grain size (*D*_50_) was calculated using GRADISTAT default settings^76^ and categorized according to the Wentworth classification^77^.

To measure dissolved oxygen in the sediment, three PME miniDO_2_T loggers were buried in the sand at depths of 5–18 cm for several hours, logging dissolved oxygen every minute. Loggers were buried horizontally and with sensor end facing into the direction of the current to ensure flow was minimally disturbed and oxygenated water was not redirected deeper into the sediment. Equilibration took approximately 30 min, and the first hour of data was discarded. Wind speed was downloaded from the Bureau of Meteorology closest weather station (St Kilda Harbour RMYS for St Kilda and Point Cook RAAF for Werribee) at 10 m.

### Dissolved methane calculations

Dissolved methane concentration was determined on the basis of the headspace methane concentration using the equilibrium concentration relationship of Wiesenburg and Guinasso 1979

$$C=\beta \left(1-{P}_{\mathrm{vp}}\right){f}_{\mathrm{G}},$$

where *C* is the equilibrium concentration of methane, *β* is the Bunsen coefficient, *P*_vp_ is the vapour pressure of the solution (atm) and *f*_G_ is the mole fraction of gas in dry atmosphere. The Bunsen coefficient is calculated usingwhere *T* is the temperature in kelvin, *S* is the salinity in ppt and A1, A2, A3, B1, B2 and B3 are empirical constants.

The per cent saturation was calculated using the difference between the dissolved methane concentration and the calculated equilibrium concentration given the atmospheric methane concentration. We used the reported variation in annual atmospheric methane concentrations to estimate uncertainty in the calculated equilibrium concentration for Northern and Southern Hemispheres^78^.

### Flux rate estimates based on in situ CH_4_ measurements

Flux rates were calculated using

$$F=k\times {K}_{0}\times \Delta C,$$

where *k* is the gas transfer velocity (m s^−1^), *K*_0_ is aqueous phase solubility determined from ref. ^79^ and Δ*C* was calculated as described above for saturation calculations.

$$k=0.251 \times {U}_{10} \times {\left(\frac{{Sc}}{660}\right)}^{-0.5},$$

where *U*_10_ is the wind speed at 10 m (m s^−1^) measured at wind station St Kilda Harbour (Royal Melbourne Yacht Squadron −37.86313444, 144.97137889). We averaged twice daily wind measurements (9 am and 3 pm) over 1 year and calculated the interquartile range for our low and high estimates, as well as the average. The Schmidt number, Sc, was calculated for seawater using the R package marelac (version 2.1.11)^80^.

### FTRs

Sand was collected for grain sizing using acrylic cores from the shallow subtidal zone and transported back to the laboratory. The top 0–5 cm were sectioned, pooled, sieved (2 mm) to remove shells and other debris and homogenized in locally collected fully oxygenated seawater. The sand was then packed into FTRs underwater and the inlet connected to a reservoir (Supplementary Fig. 11). The reservoirs contained seawater collected from the same site and were bubbled continuously with N_2_ + CO_2_ (820 ppm) to remove oxygen while maintaining the pH (anoxic treatments) or laboratory air (oxic treatments).

For site comparisons (Fig. 2a), control reservoirs contained only seawater while treatment reservoirs contained seawater + macrophyte extract (20:1 ratio, chosen as an attempt to simulate a large macrophyte accumulation event). Seawater with 10 µM methylphosphonate (MPn) was used to determine the contribution of MPn degradation to total methane production (Supplementary Fig. 2). For the repeated oxygen pulse FTR experiment (Fig. 2f), control and oxygen-exposed treatments were both circulated with a 50:1 ratio of seawater and macrophyte extract. In the oxygen-exposed treatment reservoir, the N_2_/CO_2_ gas mix was swapped for air during oxygen exposure.

Seawater was pumped through FTRs using a peristaltic pump set to 45 ml h^−1^ and not recirculated. During the oxygen pulse experiment, the flow rate was increased in all FTRs to ensure oxic conditions throughout the FTR, as oxygen demand was high and increased throughout the experiment. The rate was increased manually for both control and treatment groups and noted until outlet oxygen concentration reached approximately 50% saturation in the treatment group, and the change in flow rate was accounted for in the production rate calculations. Reservoirs were topped up with purged seawater approximately every 24 h and the macrophyte extract ratio or MPn concentration was maintained.

To enhance reproducibility across FTR experiments from different sites, a macrophyte extract was prepared as a consistent source of dissolved macrophyte metabolites. We collected mixed macrophytes from Shoreham, consisting of approximately 60% seagrass (*Amphibolis antarctica*) and the remainder as mixed seaweeds. A total of 278 g (dry weight) of mixed macrophytes was combined with 9 l of seawater and stored at 4 °C for 9 days. Solids were then strained out and the liquid extract was frozen until use.

Dissolved oxygen and pH in the reservoirs and FTR outlets were monitored with optical flow-through cell oxygen sensors (Firesting Oxygen meter, Pyroscience) and a Hach HQ40d pH meter, respectively.

Samples for CH_4_ and dissolved inorganic carbon (DIC) measurements were taken from the reservoir and FTR outlet using gas-tight glass syringes and processed as for field samples.

### Maximum theoretical flux rates based on FTRs

Calculations to normalize FTR methane production rates to wetland flux rates were performed as follows. We chose 0.5 cm as the sediment depth over which to integrate the methane production rate (rather than 2 cm as was the length of our FTRs) as this is a more conservative estimate of the advective penetration of high-substrate surface water under wave pumping in the intertidal zone^45^.

$${\mathrm{Maximum}}\; {\mathrm{rate}}\; {\mathrm{of}}\; {\mathrm{methane}}\; {\mathrm{production}}=x\approx 48\,\upmu{\mathrm{{mol}}}\times {\mathrm{cm}}_{\mathrm{sand}}^{-3}\times {\mathrm{h}}^{-1}$$

$${\mathrm{Surface}}\; {\mathrm{area}}\; {\mathrm{molar}}\; {\mathrm{flux}}\; {\mathrm{rate}}\; {\mathrm{per}}\; {\mathrm{FTR}}\; {\mathrm{integrated}}\; {\mathrm{over}}\, 0.5\,{\mathrm{cm}}$$

$$=x \times \frac{{V}_{\mathrm{FTR}}}{4}/{A}_{\mathrm{FTR}}$$

$$=24.0 \,\upmu{\mathrm{{mol}}}\times {\mathrm{cm}}^{-2}\times {\mathrm{h}}^{-1}$$

$${\mathrm{Unit}}\; {\mathrm{conversion}}\; {\mathrm{to}}\; {\mathrm{mmol}}\; {\mathrm{per}}\, {\mathrm{m}}^{2}=240\,{\mathrm{mmol}}\times {\mathrm{m}}^{-2}\times {\mathrm{h}}^{-1}$$

$${\mathrm{Mass}}\; {\mathrm{flux}}\; {\mathrm{rate}}\; {\mathrm{per}}\,{\mathrm{m}}^{2}=240\times \frac{16.04}{1,000}=3.8 {\mathrm{g}}\times {\mathrm{m}}^{-2}\times {\mathrm{h}}^{-1}.$$

### Slurries

Sand was collected and processed as described for FTRs. For each sediment slurry, 30 g sediment was transferred into a 160 ml serum vial by flushing down a funnel with 70 ml of seawater collected from the same location as the sediment. For seawater-only and seawater + macrophyte slurries, 100 ml of seawater was used, with or without 3 g wet weight of macrophyte. All treatments consisted of triplicate slurries.

Vials were crimp closed with butyl rubber stoppers and the headspace was purged for 2 min with N_2_, shaken for 2 min and then purged again to remove oxygen. To sample for CH_4_ measurements, 2 ml of He was injected into the headspace and gently agitated before a 2 ml sample was taken from the headspace and injected into a He-purged 3 ml exetainer. The samples were analysed by GC–PDHID (see ‘Chemical analysis’ section).

To examine the relative importance of sediment and macrophyte in methane production, slurries were prepared in combinations of seawater, sediment and macrophyte. Macrophytes were either brown drift algae collected from Werribee (Fig. 2b) or seagrass collected from Shoreham (*A.* *antarctica*) (Supplementary Fig. 4).

To examine the inhibition of methanogens, sediment slurries were prepared with or without 20 mM BES in the presence or absence of spirulina (dried, powdered *Arthrospira platensis* sourced from Nature’s Way, 12.5 mg per slurry). Spirulina was used for initial slurry incubations as it produces many of the same osmolytes as macroalgae^81^, as well as being easily available as a single species as a dietary supplement, increasing reproducibility.

To identify the effect of different substrates on CH_4_ production pathways, TMA, DMS, methylamine, choline, methylphosphonate or acetate was added to separate slurries. All chemicals were sourced from Sigma-Aldrich and the final concentration of the substrate in the slurries was adjusted to 100 µM. For the hydrogenotrophic pathway, hydrogen gas (H_2_) was added directly to the headspace and equilibrated to a final concentration of 340 ± 20 ppm.

Time 0 was defined as the point when oxygen was completely purged from the slurries and the substrates added, after which an initial sample was immediately taken. The incubations were run for at least 50 and up to 400 h, depending on how quickly methane was produced (that is, they were incubated until a distinct increase in methane concentration was detected in at least one treatment) with samples taken approximately every 24 h.

### Chemical analysis

#### Analysis of CH_4_ by gas chromatography

For analysis of dissolved methane samples (all in situ and FTR samples), a headspace was introduced to the 12 ml exetainer by replacing 5 ml of the seawater sample with high-purity Helium gas. Samples were then shaken vigorously for 4 min and the gas in the headspace was analysed using a GC–PDHID system (Valco Instruments Co. Inc.)^52^. The original dissolved gas concentration was calculated according to ref. ^82^ based on the Bunsen coefficients from ref. ^83^. Slurry headspace samples were injected directly.

For the pure culture experiments, the methane concentration was too high for GC–PDHID and so they were analysed by gas chromatography with a flame ionization detector. First, 100 µl samples were taken directly from the headspace with an SGE® gas-tight syringe and injected manually into a PerkinElmer Clarus 580 gas chromatograph fitted with an SGE BP20 wax column (30 m length, 0.32 mm diameter and 1 µm film thickness).

Manual triplicate five-point calibration was performed at the start of each run, along with standards and blanks at the end of each run. The calibration gases used were NATA-accredited calibration gases from Air Liquide and BOC HiQ.

#### Analysis of DIC

For analysis of DIC, a 1 ml seawater sample from FTRs and slurries was acidified using 1 M H_3_PO_4_ to convert carbonate and bicarbonate to carbon dioxide. The carbon dioxide produced was stripped from the liquid phase by bubbling with ultrapure nitrogen gas and analysed using a LI-COR nondispersive infrared analyser (Apollo SciTech). Standard reference material for three-point calibration was obtained from Scripps Institute of Oceanography, University of California, San Diego.

#### Radon analysis for groundwater input

Radon (^222^Rn) concentration was analysed as a tracer for groundwater using a ^222^Rn in-air monitor (RAD7, Durridge Company). First, 1 l of the water sample was collected in Schott bottles and capped underwater where possible to eliminate bubbles. The samples were analysed in the laboratory within 24 h of collection. For analysis, 500 ml sample was degassed for 5 min into a closed-air loop of known volume and was counted for 2 h. The concentrations were corrected for salinity, temperature and decay time loss to accurately represent in situ concentrations.

#### Metabolomic analysis by LC–MS

The macrophyte extract used in FTR experiments was analysed for methylated compounds using targeted and untargeted liquid chromatography–mass spectrometry (LC–MS). First, 160 µl of extraction solvent (1:3 chloroform:methanol) with 2 µM CHAPS, CAPS, PIPES and TRIS) internal standard was added to a 20 µl sample of macrophyte extract, vortexed and sonicated in an ice bath for 10 min and centrifuged at max speed for 10 min at 4 °C to crash out proteins and lipids. The supernatant was then transferred and analysed in triplicate by hydrophilic interaction LC−MS. Briefly, an iHILIC-(P) classic, HILIC column (PEEK,150 × 4.6 mm, 5μm, 200 Å) with a 1290 inline filter, 0.3 μm (Agilent) as a guard column was maintained at 25 °C with a gradient elution of 20 mM ammonium carbonate (A) and acetonitrile (B) (linear gradient time and percentage B as follows: 0 min 80%, 15 min 50%, 18 min 5%, 21 min 5%, 24 min 80% and 32 min 80%) on a Vanquish Horizon (Thermo Fisher Scientific). The flow rate was maintained at 500 μl min^−1^. Samples were kept at 6 °C in the autosampler and 10 μl was injected for analysis. Mass spectrometry was performed on a Q-Exactive Plus Orbitrap (Thermo Fisher Scientific) in MS1 polarity switching mode. The instrument was operated at 70,000 resolution with the following conditions: electrospray ionization voltage was 4 kV in positive and −3.5 kV in negative mode, capillary temperature of 300 °C, sheath gas of 50, Aux gas of 20, sweep gas of 2 and probe temperature of 120 °C).

A seven-point calibration curve was used for the quantification of TMA, TMAO, DMSP, DMS and choline, with concentrations selected on the basis of a pilot study of similar samples, therefore all measured concentrations fell within the calibration range. Peak integration of target metabolites was performed in Skyline 24.1. (refs. ^84–86^).

### DNA extraction and sequencing

Samples for metagenomic analysis were subsampled from sediment cores collected for FTR experiments (0–5 cm depth). Additional samples for metagenomic analysis were subsampled from Shoreham FTR sediment at the end of the experiment (*T* = ~60 h).

Total community DNA was extracted from 0.25 g of sediment using the Qiagen DNeasy PowerSoil Pro extraction kit according to the manufacturer’s instructions. The DNA yield was measured by a Qubit 2.0 Fluorometer (Invitrogen). Shotgun sequencing was conducted by Micromon Genomics using DNBSEQ-G400 at 10 Gb depth.

### Metagenomic analysis

The Metaphor pipeline^87^ was employed for read quality control and assembly. Specifically, raw reads from the eight metagenome libraries underwent quality control by trimming primers and adaptors, removal of artefacts and low-quality reads using fastp^88^ with the parameters length_required: 50, cut_mean_quality: 30 and extra:–detect_adapter_for_pe.

To estimate the metabolic capabilities of the microbial communities, quality-filtered short reads were searched against a custom made protein database and Kyoto Encyclopedia of Genes and Genomes (KEGG) database^89^ covering methane cycling genes. DIAMOND v.0.9.31 searches^90^ were conducted with a query coverage of 80% and identity threshold of 50% for the genes methyl coenzyme M reductase (*mcrA*) and alpha-d-ribose 1-methylphosphonate 5-phosphate C-P lyase (*phnJ*) and an identity threshold of 70% for *Acidovorax* sp. strain MeA-13 aspartate aminotransferase (*aat*). Read counts were normalized to reads per kilobase per million and further normalized against a mean reads per kilobase per million value estimated from 14 single-copy ribosomal marker genes to infer the percentage of the community encoding each gene.

Quality-filtered reads were also co-assembled using MEGAHIT v1.2.9 (ref. ^91^) with default settings. Contigs shorter than 1,000 bp were discarded. Open reading frames were predicted using Prodigal v2.6.2.9 (ref. ^92^), then annotated using homology-based searches using DIAMOND blastp^93^ against a custom protein database as described^94^. Only *mcrA* sequences with a query coverage of at least 80% and a percentage identity of at least 50% were retained. Full scripts and settings are provided in ref. ^95^.

### Methanogen isolation and whole-genome sequencing

Isolation experiments were undertaken from the surface 5 cm of sediment of two highly macrophyte-impacted sandy beaches on Avernakø and Shoreham, collected on 10 July 2023 and 22 January 2024, respectively. Methylotrophic methanogens were enriched in slurries prepared as above, with the addition of 1 mM TMA and incubation at 15 °C (Denmark) or 19–21 °C (Australia) for 4 weeks. A methanogen was then isolated from each enrichment by three rounds of dilution to extinction in modified DSMZ 141c *Methanococcoides* medium (modifications were the addition of 0.5 mg l^−1^ sodium resazurin, ampicillin 100 mg l^−1^ and kanamycin 200 mg l^−1^, and the exclusion of yeast extract, sodium acetate and trypticase peptone). The purity of the isolate was ensured by adding antibiotics to a methanogen-selective isolation media. The growth of the methanogenic strains was monitored by OD_600_ measurements, optical microscopy and methane production, and the resulting strains referred to as DA (Denmark) and SH (Australia). Purity was then confirmed through epifluorescence microscopy, targeting the F_420_ autofluorescence typical of methanogens, along with whole-genome sequencing.

For whole-genome sequencing, DNA was extracted using the DNeasy PowerSoil Pro kit according to the manufacturer’s instructions. For strain DA, DNA concentration and purity were measured with the Qubit dsDNA HS Assay kit (Thermo Fisher Scientific) and NanoDrop One, respectively. DNA size distributions were evaluated using the Genomic DNA ScreenTapes on Agilent Tapestation 4200. A barcoded SQK-NBD114.95 DNA library was prepared (Oxford Nanopore Technologies) and loaded onto a primed FLO-PRO114M (R10.4.1) flow cell and sequenced on a PromethION P24 device. For strain SH, next-generation sequencing library preparations were constructed following the manufacturer’s protocol (VAHTS Universal Plus DNA Library Prep kit for Illumina V2). Libraries with different indexes were multiplexed and loaded on an Illumina NovaSeq 6000 instrument according to the manufacturer’s instructions (Illumina). Sequencing was carried out using a 2× 150 paired-end configuration. Image analysis and base calling were conducted by the NovaSeq Control Software + RTA 3 (Illumina) on the NovaSeq 6000 instrument.

The CoverM v0.6.1 (ref. ^96^) ‘genome’ was used to calculate the relative abundance of the methanogen isolates in the metagenomes.

### Oxygen pulse exposure experiment

Isolate cultures were grown on TMA to stationary phase in 20 ml crimp-top vials as for isolation, but with contactless autoclavable oxygen sensor spots inserted at the bottom (PyroScience OXSP5 oxygen sensor spots, calibrated according to the manufacturer’s instructions). At the beginning of the experiment, approximately 10 ml of laboratory air was injected into the headspace of treatment vials, with an outlet needle inserted to prevent overpressure, and the vials shaken to equilibrium. Oxygen readings were taken every 5 s in all oxygen exposure treatment vials plus one no oxygen exposure vial (as the maximum number of channels measuring oxygen concurrently is four). After 30 min, all vials were flushed with helium to remove oxygen and residual methane. As soon as the dissolved oxygen readings in oxygen exposure treatments were stable at <0.01 mg l^−1^ and no pink (oxidized) resazurin was visible, 100 µl of sterile anoxic 500 g l^−1^ TMA was then injected into all vials (including the media control) and methane measurements began. Methane in the headspace was analysed approximately every hour for 5 h. For strain SH, control and treatment were prepared in duplicate, and strain DA in triplicate.

### Phylogenetic tree building and visualization

We used GTDB-tk^97^ to align the genomes of the two isolates with 71 reference methanogens genomes, whereas we used Muscle^98^ to align the three *mcrA* contigs with 196 and 31 *mcrA* reference sequences for producing the alignments used to generate the supplementary and main *mcrA* phylogenetic trees, respectively. Phylogenetic trees were generated using IQ-TREE v2.2.2.6 (refs. ^99,100^) and 1,000 ultrafast bootstraps. Genome tree was generated with model LG + F + I + R6, *mcrA* supplementary tree alignment was generated with model LG + F + I + R5; and *mcrA* main tree alignment was generated with model LG + I + G4. We plotted the phylogenetic trees with genome statistics using iTOL v6 (ref. ^101^). Full scripts and settings are provided in ref. ^95^.

## Online content

Any methods, additional references, Nature Portfolio reporting summaries, source data, extended data, supplementary information, acknowledgements, peer review information; details of author contributions and competing interests; and statements of data and code availability are available at 10.1038/s41561-025-01768-3.

## Supplementary information

Supplementary Information Supplementary Figs. 1–11 and Tables 1–4.

## Data Availability

The sequence data generated and analysed during the current study, including metagenomes, isolate genomes and contigs, are freely available at NCBI Sequence Read Archive BioProject ID: PRJNA1165813 at https://www.ncbi.nlm.nih.gov/bioproject/PRJNA1165813.

## Code availability

All scripts for bioinformatic analysis, supplementary trees and tree files are available via GitHub at https://github.com/GreeningLab/Sand-methanogen-manuscript (ref. ^95^).
