# Supplementary material for: Coastal methane emissions driven by aerotolerant methanogens using seaweed and seagrass metabolites
Source: Nat Geosci. 2025 Aug 7;18(9):854–61. doi: 10.1038/s41561-025-01768-3 (PMC12422968; doi:10.1038/s41561-025-01768-3)
Supplement: Supplementary file 1 — Supplementary Figs. 1–11 and Tables 1–4. [file 41561_2025_1768_MOESM1_ESM.pdf]

nature geoscience

Supplementary information

https://doi.org/10.1038/s41561-025-01768-3

Coastal methane emissions driven by aerotolerant methanogens using seaweed and seagrass metabolites

In the format provided by the authors and unedited

Table of contents

**Supplementary Figures:**	**Page**
**Figure S1.** Dissolved oxygen profile of sediments	2
**Figure S2.** Methylphosphonate treated flow through reactors	2
**Figure S3.** Methane, dissolved oxygen and dissolved inorganic carbon production rate in Shoreham flow through reactors	3
**Figure S4.** Slurry experiments with combinations of seawater, seagrass, and surface sediment, with and without methanogenesis inhibitor	3
**Figure S5.** Photos of all sampling sites in the study	4
**Figure S6.** Sediment core from Avernakø East	5
**Figure S7.** Sediment cores from St Kilda B	6
**Figure S8.** Sediment core from Shoreham	6
**Figure S9.** Sediment core from Werribee	7
**Figure S10.** Photos of methanogen cultures	8
**Figure S11.** Diagram of lab flow through reactor setup and closeup	9

**Supplementary Tables:**	
**Table S1.** Field site descriptions, grain size, and macrophyte accumulation characteristics	9
**Table S2.** Field site locations, average surface water methane concentrations, and calculated percent saturation	10-11
**Table S3. Pathways of methane cycling in coastal marine environments**	12
**Table S4. Relative gene abundance of** potential methane producing enzymes	12

Other Supplementary Materials for this manuscript include the following:

Scripts and mcrA sequence affiliation file accessible at https://github.com/GreeningLab/Sand-methanogen-manuscript

Sequence data (metagenomes and isolate genomes) available at NCBI Sequence Read Archive Accession: PRJNA1165813

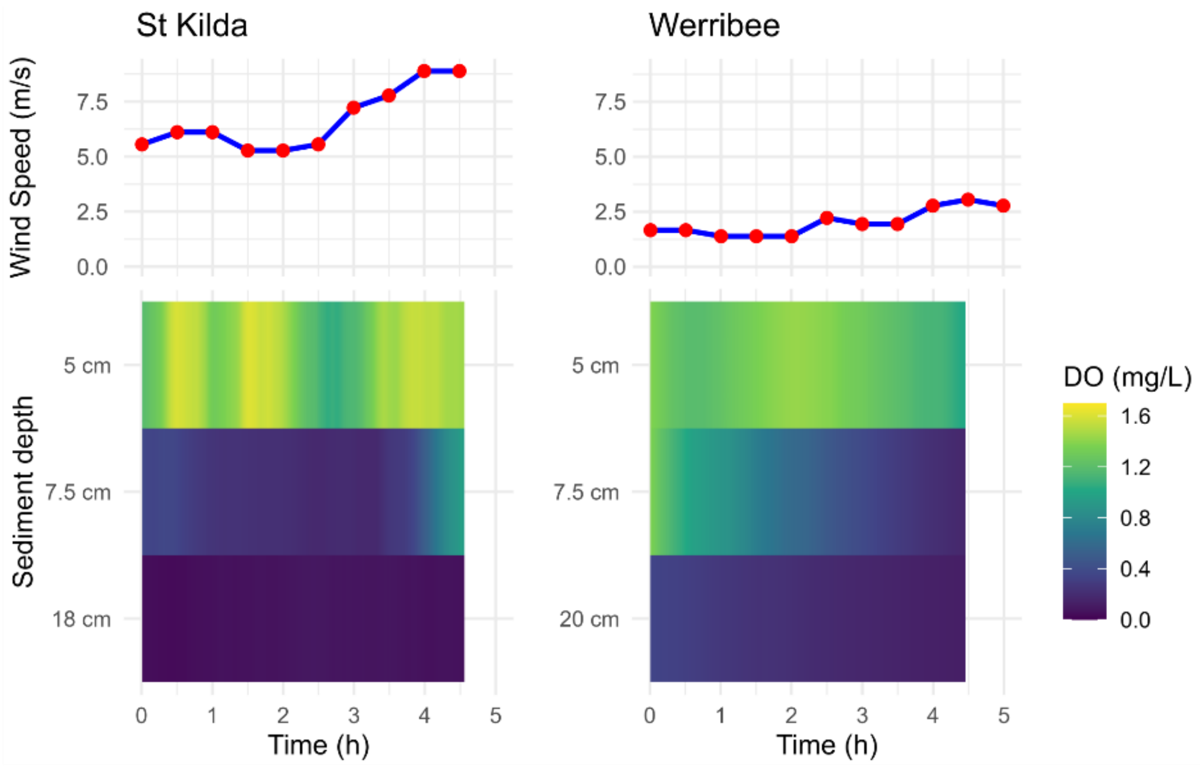

Figure S1. Dissolved oxygen profile of sediments and windspeed at St Kilda B and Werribee.

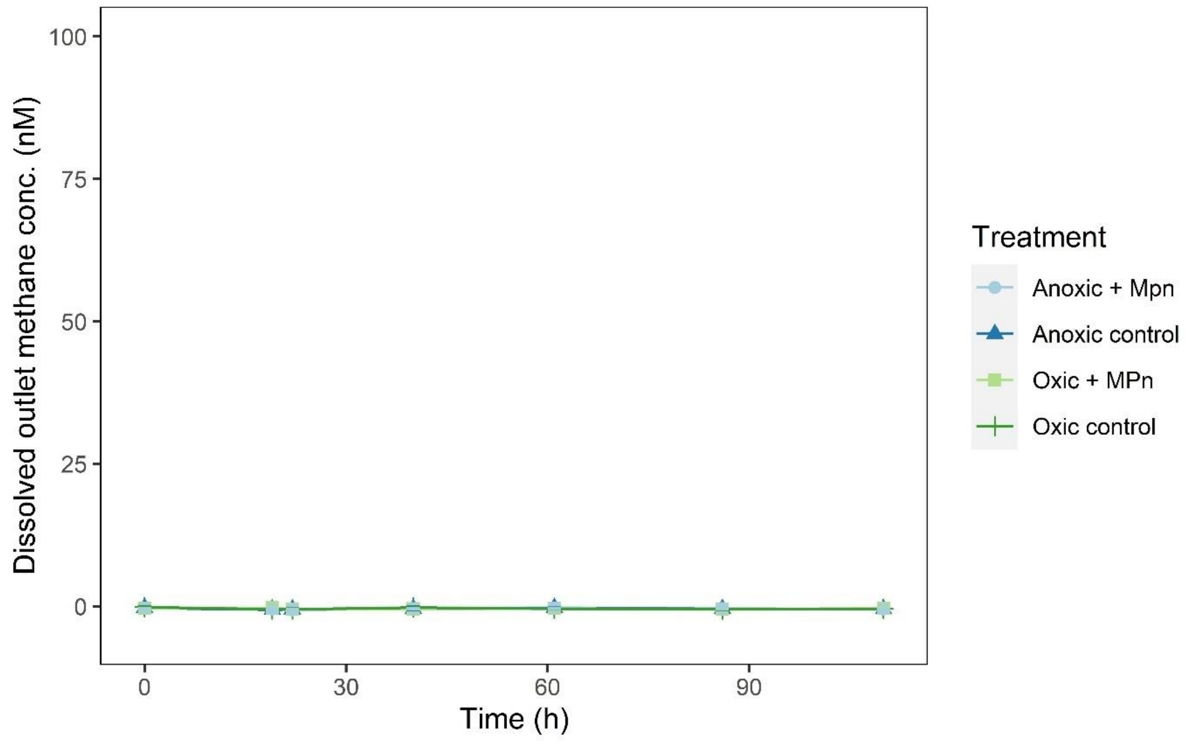

Figure S2. Oxic and anoxic flow through reactors (surface 0-5 cm Werribee sediments collected 14/10/2021) with and without addition of methylphosphonate (MPn, 10 µM) in seawater reservoir.

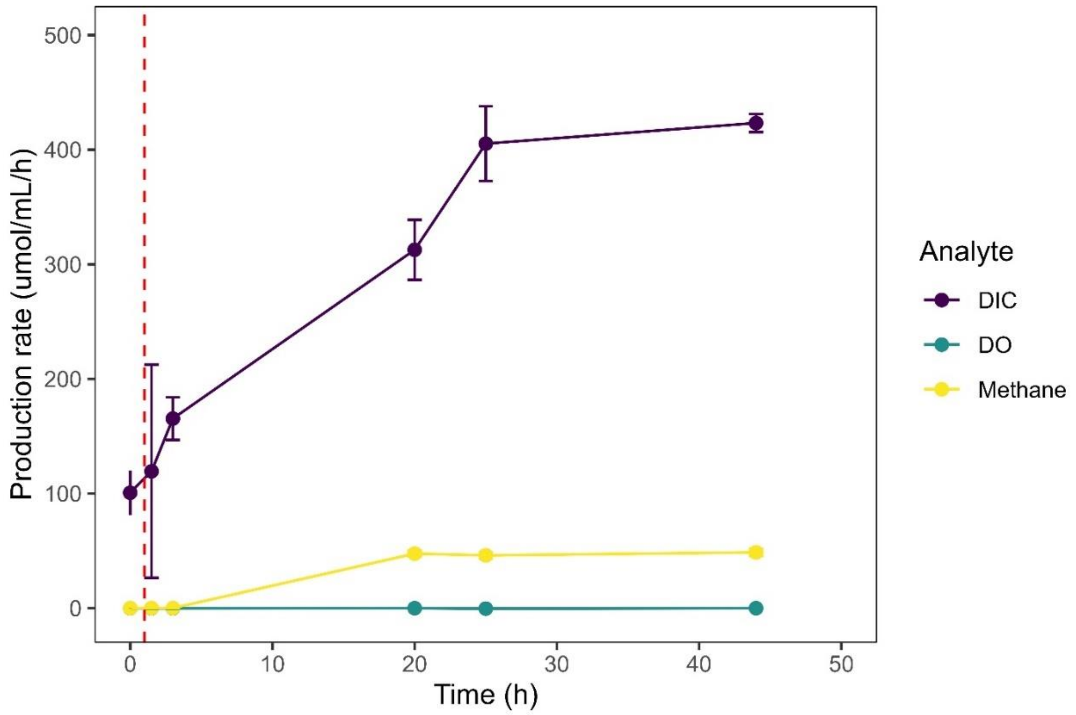

Figure S3. Methane, dissolved oxygen (DO) and dissolved inorganic carbon production rate in Shoreham FTRs with macrophyte extract, normalized to µmol/mL$_{sediment}$/h. Error bars represent one standard deviation from the mean, n = 3. Note error bars for methane and dissolved oxygen (DO) are present but too small to see at the given scale.

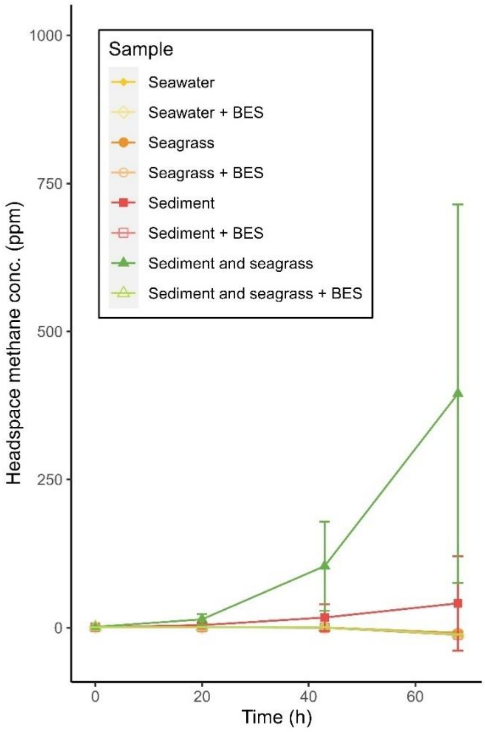

Figure S4. Slurry experiments with combinations of seawater, seagrass, and surface sediment (0-5 cm depth at Shoreham) as well as specific archaeal methanogenesis inhibitor 2-bromoethane sulfonate (BES) 20 mM. Error bars represent one standard deviation from mean, n = 3.

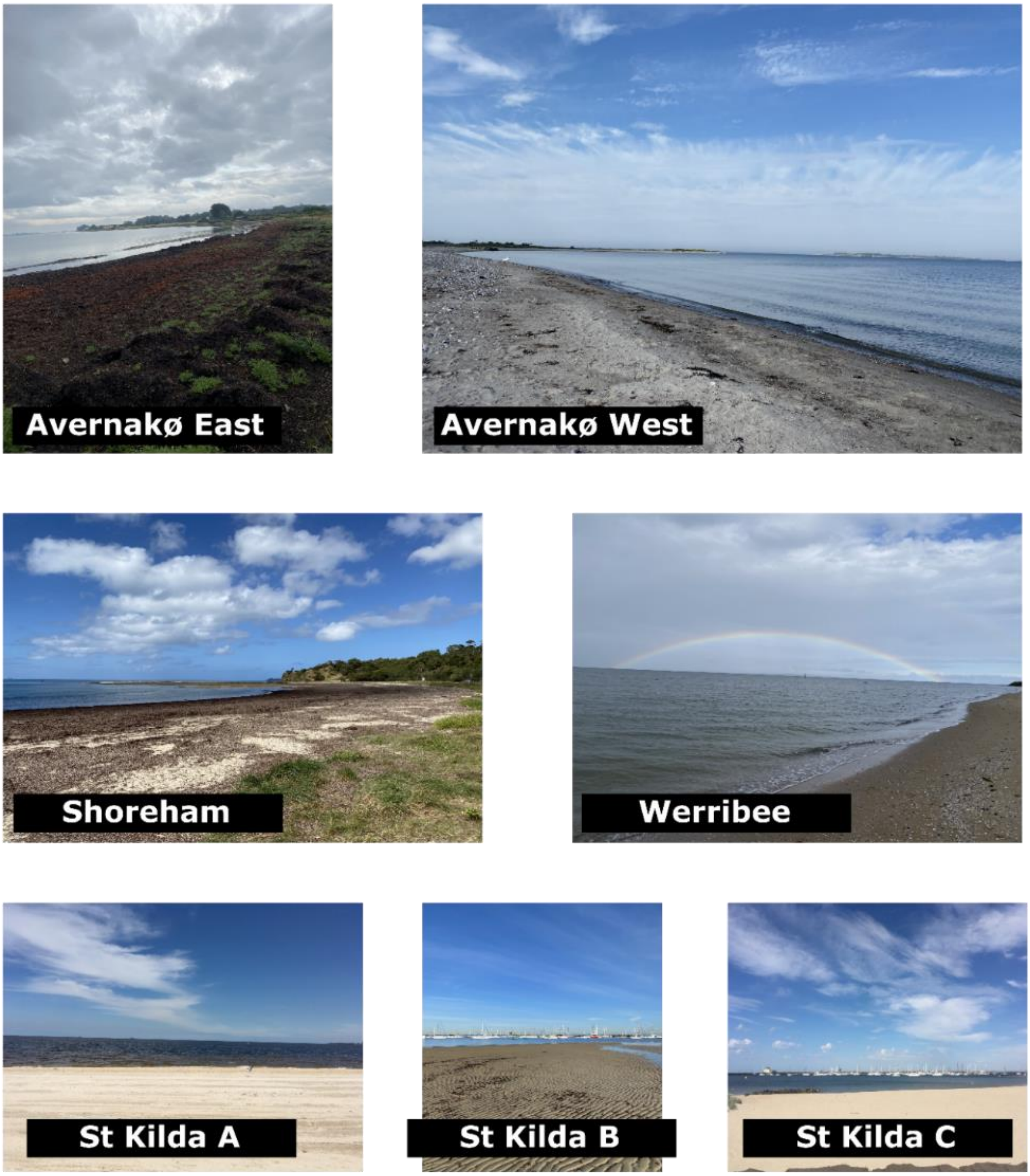

Figure S5. Photos of all sampling sites in the study.

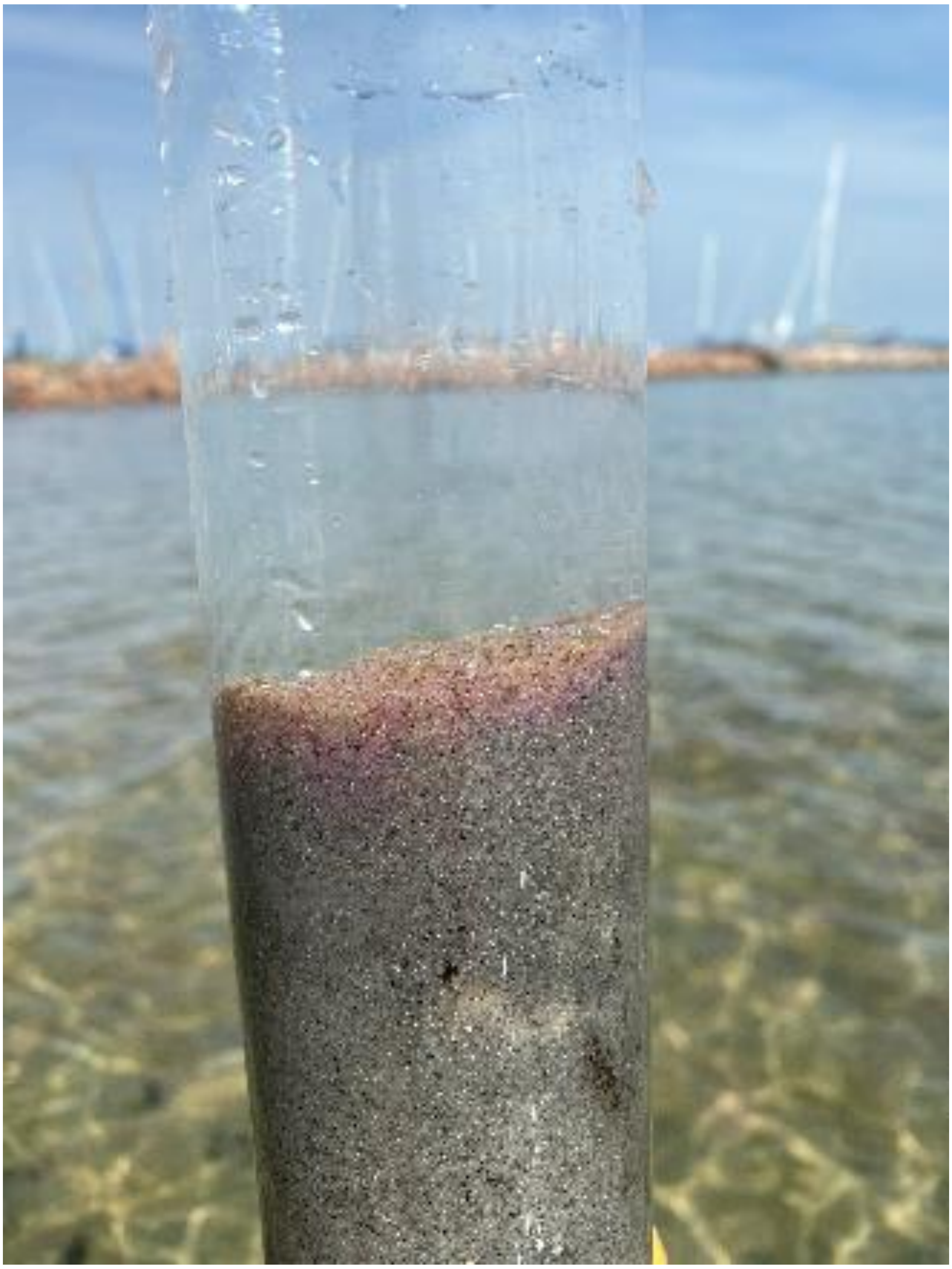

Figure S6. Sediment core from Avernakø East. Surface 5 cm used for isolation of DA as well as slurry experiment (main text Fig. 1G).

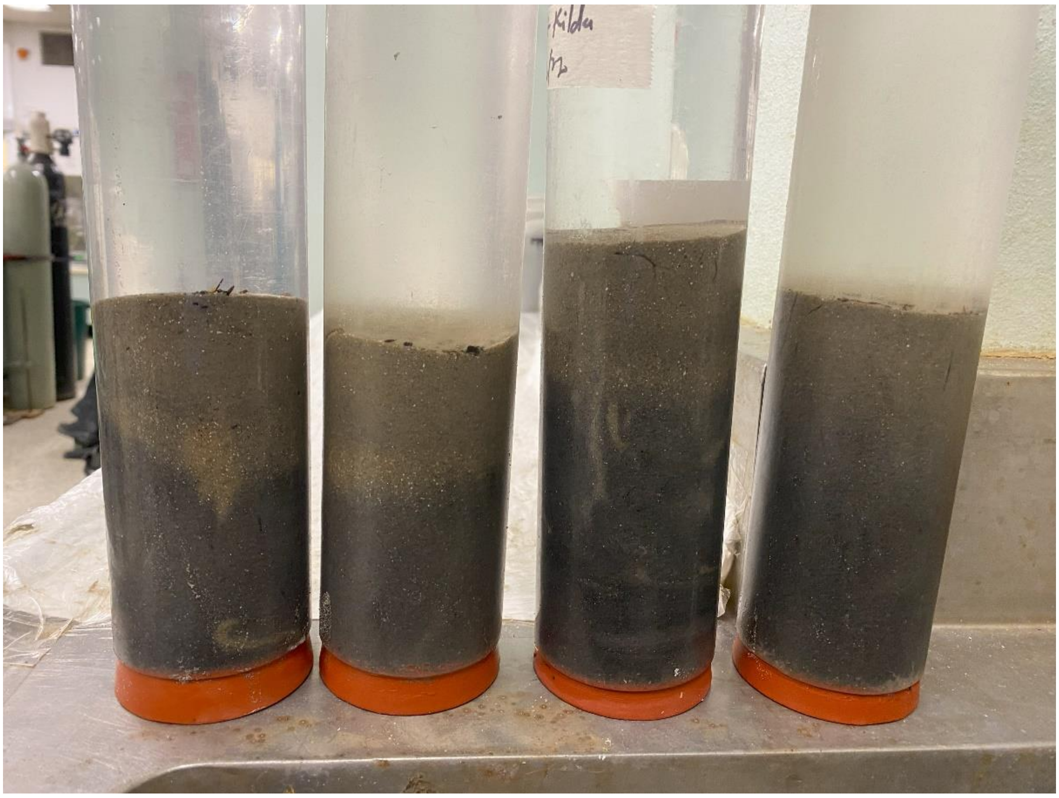

Figure S7. Sediment cores from St Kilda B. Used for FTR experiment (Fig. 1C).

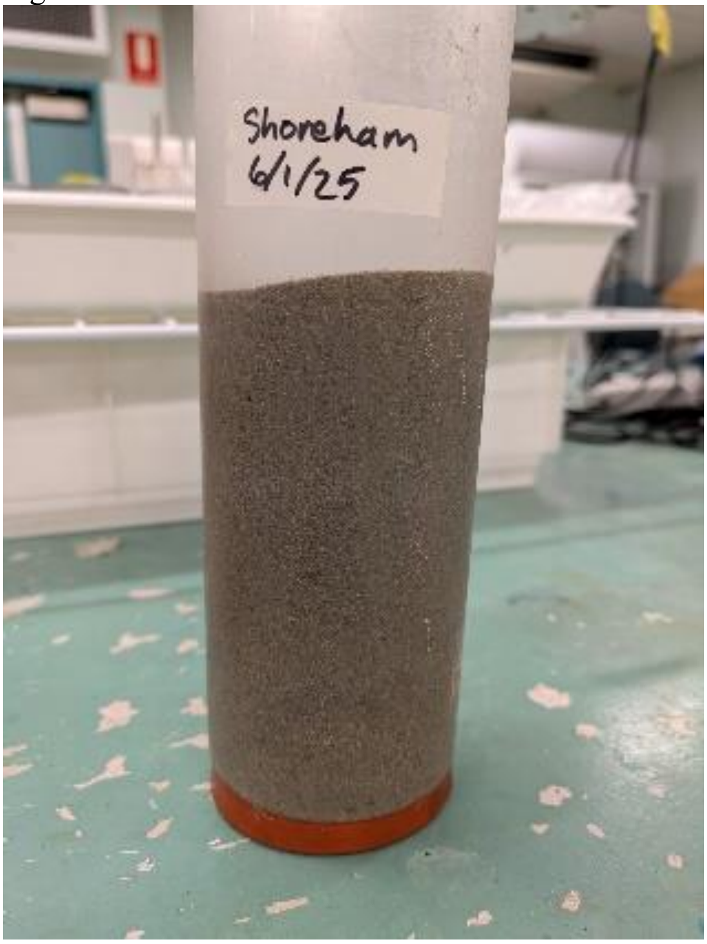

Figure S8. Sediment core from Shoreham, used for isolation of SH, slurry experiments (Fig.1F) and FTR experiments (Fig. 1C, H)

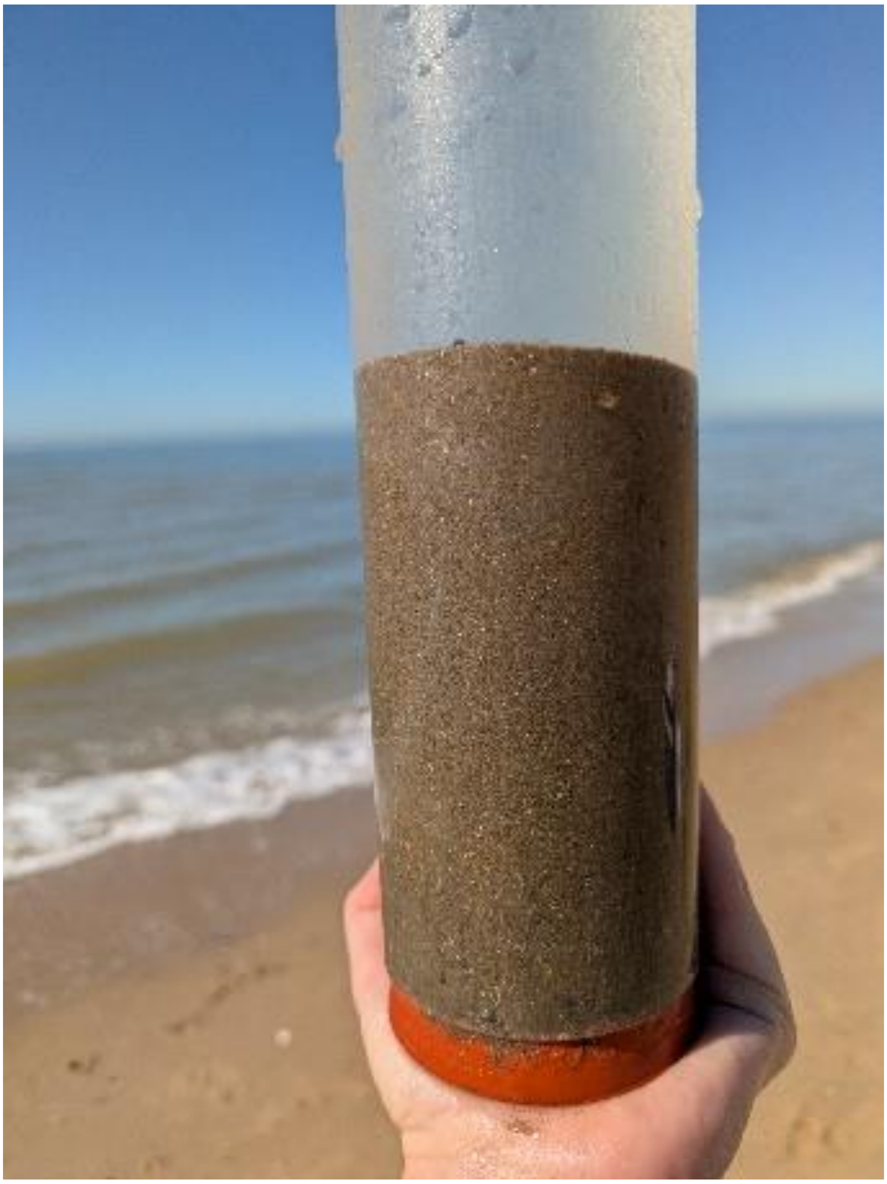

Figure S9. Sediment core from Werribee. Used for slurry experiments (Fig. 1D, E) and FTR experiment (Fig. 1C).

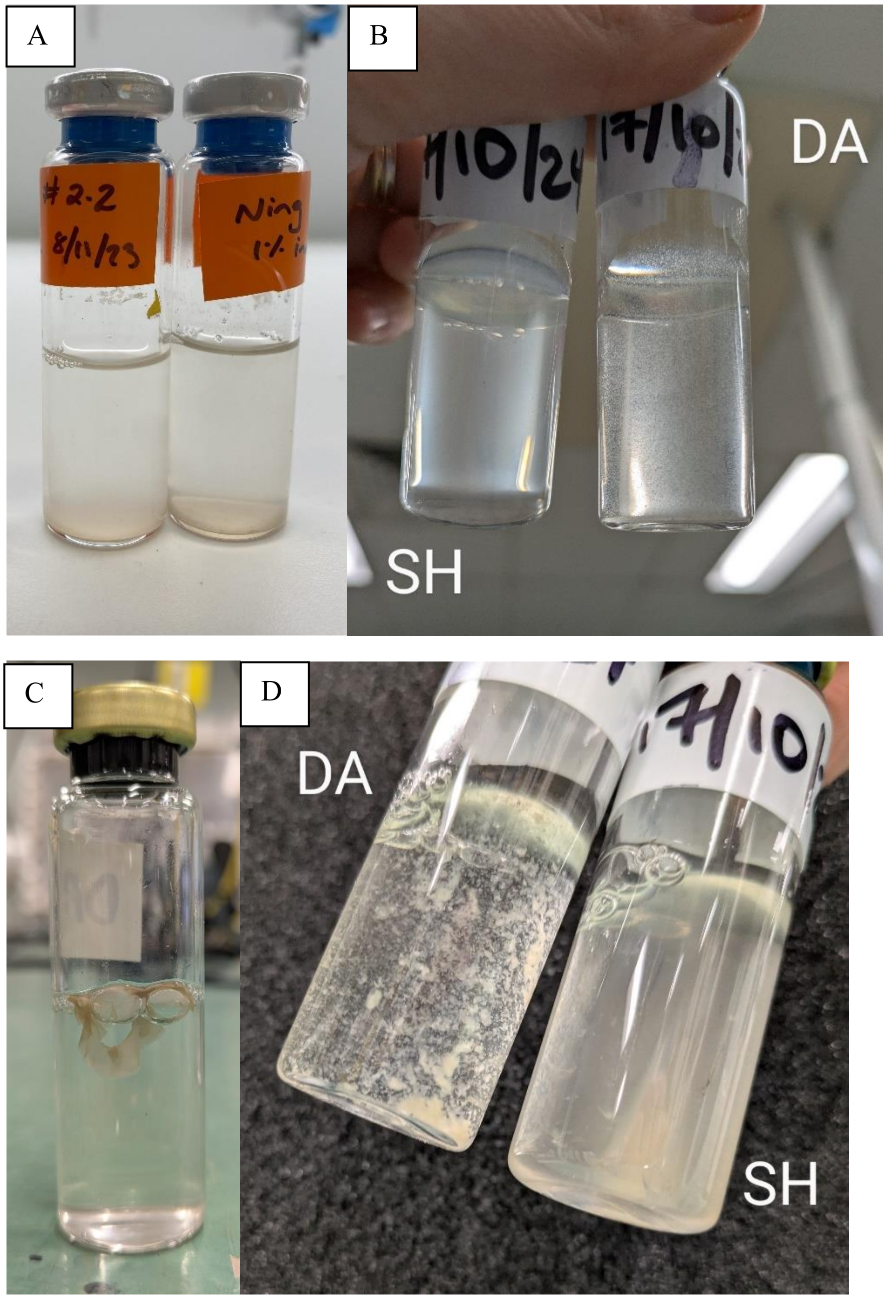

Figure S10. Photos of methanogen cultures (A) DA exhibiting uniform growth (B) SH (uniform growth) and DA forming small clumps (C) DA having formed a surface film (D) SH (uniform growth) and DA forming large clumps.

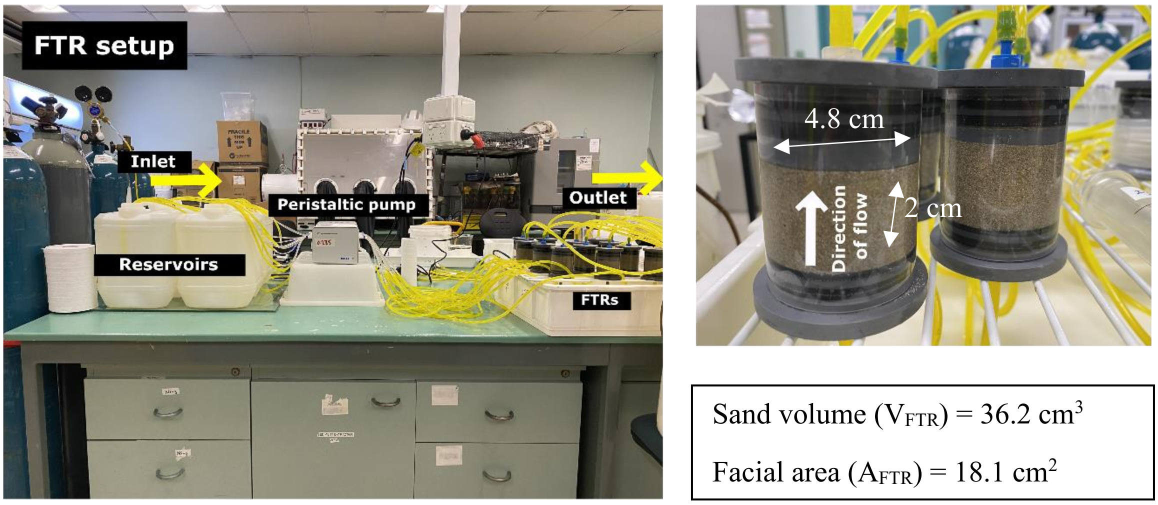

Figure S11. Diagram of lab flow through reactor (FTR) setup (left) and close up of FTR (right) including dimensions of sand cavity.

Table S1. Field site descriptions, median grain size (D_{50}) and Udden-Wentworth grain size scale classification and macrophyte accumulation characteristics. * indicates values previously reported in Chen et al. 2022

Site	Median grain size (D_{50}) and classification	Description and macrophyte accumulation characteristics
**Avernakø**	Not measured	Two beaches on East (Av 1, 2, 3) and West (Av 4, 5) sides of Avernakø boat harbor. Avernakø East had large accumulations of macrophyte (very degraded so identification was not possible) while Avernakø West was almost bare, with small isolated pieces of ulva
**St Kilda A**	0.443 mm*, medium sand	Exposed, high energy beach (relative to other St Kilda sites) with no visible macrophyte accumulation.
**St Kilda B**	0.295 mm*, medium sand	Sandy beach at entrance to boat harbor and partially protected by a breakwater. Small isolated pieces of seaweed (mostly ulva)
**St Kilda C**	0.217 mm*, fine sand	Sandy beach in boat harbor and highly protected by a breakwater. Contains very. Very small isolated pieces of seaweed (mostly ulva)
**Shoreham**	0.264 mm, medium sand	Large mounds of macrophyte biomass partially submerged in surf. Approximately 60% seagrass (primarily *Amphibolis antarctica*) with remainder mixed seaweeds including ulva, kelp, and filamentous red species.
**Werribee**	0.277 mm, medium sand	A history of large seasonal deposits of drift algae (brown). Radon/methane correlation study conducted at the tail end of one of these blooms with one small remaining patch. All other experiments (FTRs and slurries) conducted when no seaweed was present.

Table S2. Field site locations, average surface water methane concentrations (n=3), and calculated percent saturation. Av = Avernakø, Denmark. SK = St Kilda (A-C), Sh = Shoreham, WSB = Werribee Southern Beach, WNB = Werribee Northern Beach, WH = Werribee Harbor, Australia. Numbers refer to subsites, usually approx. 20 meters apart forming a transect along the beach. Calculated % saturation uses equilibrium concentration calculated using 1922 ppb average annual atmospheric methane concentration (*1*) and equilibrium solubility based on Yamamoto et al. 1976 and Wiesenburg and Guinasso 1979 (*2*, *3*).

**Site**	**Date sampled (DD/MM/YY)**	**Lat.**	**Long.**	**Water temp (˚C)**	**Salinity (‰)**	**Average dissolved CH_4 conc. (nM) ± SD**	**Equilibrium CH_4 conc. (nM)***	**%sat.**
Av1	10/07/23	55.03896	10.25353	11.8	21.0	2100 ± 300	2.34 ± 0.04	90 000 ± 12 000
Av2	10/07/23	55.03853	10.2535	-	-	4200 ± 200		180 000 ± 9000
Av3	10/07/23	55.03799	10.25357	-	-	4400 ± 200		189 000 ± 7000
Av4	10/07/23	55.03978	10.25001	-	-	110 ± 30		4700 ± 1300
Av5	10/07/23	55.03954	10.24972	-	-	43 ± 9		1800 ± 400
SKA1	06/10/22	-37.8516	144.9553	15.1	37.2	10.1 ± 0.9	2.36 ± 0.02	430 ± 40
SKA2	06/10/22	-37.8515	144.955	-	-	9.0 ± 0.7		380 ± 30
SKA3	06/10/22	-37.8514	144.9546	-	-	9.6 ± 1.4		410 ± 60
SKB1	06/10/22	-37.8611	144.9662	-	-	144 ± 5		6200 ± 200
SKB2	06/10/22	-37.8605	144.9655	-	-	106.7 ± 0.9		4550 ± 50
SKB3	06/10/22	-37.8605	144.9655	-	-	278 ± 5		11 800 ± 200
SKC1	06/10/22	-37.8619	144.9699	-	-	66.5 ± 0.9		2840 ± 40
SKC2	06/10/22	-37.8616	144.9694	-	-	82.8 ± 1.4		3540 ± 60
SKC3	06/10/22	-37.8621	144.9693	-	-	17.0 ± 0.3		724 ± 13
Sh1	13/09/22	-38.4343	145.0477	12.9	35.8	200.9 ± 0.4	2.50 ± 0.02	8060 ± 50
Sh2	13/09/22	-38.4349	145.0476	-	-	226 ± 7		9100 ± 300
Sh3	13/09/22	-38.435	145.0473	-	-	114 ± 3		4580 ± 130
Sh4	13/09/22	-38.4352	145.047	-	-	67.7 ± 0.6		2710 ± 30
Sh5	13/09/22	-38.4353	145.0468	-	-	119 ± 13		4800 ± 500
WSB1	18/10/22	-37.9705	144.7044	18.3	32.6	29.0 ± 1.1	2.27 ± 0.02	5000 ± 200
WSB2	18/10/22	-37.9707	144.7041	-	-	23.8 ± 1.4		6700 ± 200
WSB3	18/10/22	-37.9708	144.7038	-	-	24.4 ±0.5		7100 ± 200

WSB4	18/10/22	-37.9711	144.7034	-	-	32.7 ± 0.8		7100 ± 200
WSB5	18/10/22	-37.9713	144.7029	-	-	60 ± 2		6400 ± 200
WNB1	18/10/22	-37.96326	144.71112	-	-	101 ± 2		4500 ± 100
WNB2	18/10/22	-37.96347	144.71078	-	-	95 ± 2		4200 ± 100
WNB3	18/10/22	-37.96384	144.71036	-	-	180 ± 80		8000 ± 3000
WH1	18/10/22	-37.96515	144.70836	-	-	240 ± 90		49 000 ± 4000
WH2	18/10/22	-37.96482	144.70885	-	-	2400 ± 800		70 000 ± 40 000
WH3	18/10/22	-37.96457	144.70950	-	-	600 ± 300		27 000 ± 12 000

*The error provided is based on annual variation in atmospheric methane concentration of approximately 60 ppb in the Northern hemisphere and 30 ppb in the Southern hemisphere (*4*). While local methane concentration could vary, it is unlikely to be significant based on the distance of sampling sites from any large sources of fugitive emissions such as coal seams, dairy farms or landfills. We investigated the possibility that Werribee may be slightly impacted due to proximity to a wastewater treatment plant (WWTP), however a study of a similar WWTP in New South Wales, Australia, showed typical plumes from these sites to be so narrow that while ambient concentrations near biosolid lagoons could be up to 18 ppm, in other parts of the same facility concentrations hover around atmospheric background concentrations and therefore this plume is unlikely to significantly affect the study site 1 km from the closest point of the WWTP (*5*).

Table S3. Pathways of methane cycling in coastal marine environments. TMA = trimethylamine, DMA = dimethylamine, MeA = methylamine, DMS = dimethyl sulfide.

Pathway	Substrate/s	Marker gene/s	Organism	Conditions	Ref.
MPn degradation	Methylphosphonate	*phnJ*	Bacteria	Oxic, P-limited (oligotrophic) water column	(*6*)
Aerobic bacterial methane synthesis	Methylamine	*aat*	Bacteria	Oxic	(*7*)
Methylotrophic methanogenesis	Methylated amines (TMA, DMA, MeA), methylated sulfides (DMS, methane thiol), methanol	*mcrA*	Archaea	Anoxic sediments or micro anoxic niche*	(*8*)
Aceticlastic methanogenesis	Acetate	*mcrA*	Archaea	Anoxic, low sulfate concentration sediments or micro anoxic niche*	(*8*)
Hydrogenotrophic methanogenesis	H_2 or formate and CO_2 or carbonate	*mcrA*	Archaea	Anoxic, low sulfate concentration sediments or micro anoxic niche*	(*8*)
Algal	Carbonate	Unknown	Cyanobacteria	Oxic water column	(*9*)

*E.g. zooplankton gut or sinking particle

Table S4. Relative gene abundance of C-P lyase (*phnJ*) and PLP aspartate aminotransferase (*aat*) and Methyl coenzyme M reductase alpha subunit (*mcrA*).

Gene/Sample	ShA	ShB	ShS	SKB0	WSB
(*phnJ*) alpha-D-ribose 1-methylphosphonate 5-phosphate C-P lyase [EC:4.7.1.1]	4.190	1.788	1.621	4.775	1.464
(*aat*) *Acidovorax* sp. strain MeA-13 aspartate aminotransferase	0.962	0.671	0.836	1.330	1.011
(*mcrA*) Methyl coenzyme M reductase alpha subunit	0.022	0.475	0.075	0.123	0.053
